# Automating PINN-based kinematic resolution of robotic joints using robotic process automation frameworks

**DOI:** 10.3389/frobt.2025.1752595

**Published:** 2026-01-13

**Authors:** Parth Agrawal, Pavithra Sekar, Kush Kumar Kushwaha

**Affiliations:** School of Computer Science and Engineering, Vellore Institute of Technology, Chennai, Tamil Nadu, India

**Keywords:** motion planning, physics-informed neural networks, rigid robotic joint motion, robot Operating system (ROS), RPA, robot navigation

## Abstract

This paper explores the integration of Physics-Informed Neural Networks (PINNs) and Robot Process Automation (RPA) tools in modeling and controlling rigid robotic joint motion. PINNs, which integrate physical laws with neural networks, offer a promising solution for solving both forward and inverse problems in robotics, while RPA tools provide the means to automate and streamline these processes. The study discusses various PINN techniques, including Extended PINNs, Hybrid PINNs, and Minimized Loss techniques, developed to address issues such as high training costs and slow convergence rates. By combining these advanced PINN approaches with RPA tools, the research aims to enhance the precision and efficiency of robot control, motion planning, and process automation, particularly in non-linear and dynamic coupling situations. We also examine PDE-Inspired PINNs for motion planning in robot navigation and manipulation by integrating it with ROS using the RPA tool itself for coordinating joints and angle movements, and exploring how RPA can facilitate the implementation of these models in real-world scenarios.

## 1 Introduction

Robotic technology is today expanding due to the demand for higher level control and optimization methods that would allow the robot to work and move with high precision within a given environment. This paper revolves around Robotics and Automation with substantial attention to the use of Physics-Informed Neural Networks (PINNs) for modeling and controlling rigid robotic joint motion using environmental data. Since newly developed robotic systems become more sophisticated, it is essential to provide methods for analyzing those multi-joint systems more precisely, especially in situations where external effects strongly affect the systems. One promising solution to this challenge is the use of PINNs, which incorporates physical laws with neural networks for modeling and solving forward and inverse problems in robotics. As it has been mentioned before, PINNs draw much attention in recent years because of their effectiveness in solving complicated differential equations, which widely exist in many engineering and science fields. These neural networks find the most use in solving forward problems, where the given factors determine the behavior of the given system, as well as for solving inverse problems, where the parameters of the given system are determined from the observed behavior. PINNs have been applied in various areas including fluid dynamics, heat transfer, and material science (Cuomo et al., 2022) and have proven their ability to capture complex physical phenomena with great accuracy. In these applications, PINNs have been used in modeling fluid dynamics, predicting temperature fields, and analyzing material behavior, among others. The multiple uses of PINNs in the solution of PDEs suggest that it could be useful in addressing complexities in the behavior of joint dynamics in robots.

Nonetheless, some common critical issues such as high training costs and slow convergence rate, are somewhat regarded as the drawbacks of PINNs. To address these issues, researchers have developed various PINN techniques, which can be broadly categorized into three types: Extended PINNs, Hybrid PINNs, and Minimized Loss techniques (Lawal et al., 2022). The basic framework of PINNs can be extended in the form of Extended PINNs where modification is made to the neural network structure for a better capture of the dynamics or Hybrid PINNs which use a combination of traditional numerical methods and the neural network methodologies. The Techniques under Minimized Loss usually aim to improve the convergence or the rate and frequency at which the loss function is computed. Some of this progress made in the methodologies of PINN have been vital in enhancing the applicability of the approach in real-time, or in any field where computational power may be a constraint.

However, a significant barrier remains in the operationalization of these advanced models within real-world robotic systems. The transition from a simulated PINN model to a physically deployed, automated workflow is often hampered by a ‘deployment gap,’ where significant manual intervention is required to interface the model with robot control frameworks like ROS and manage the data pipeline. This research addresses this gap by proposing a novel methodological framework where Robotic Process Automation (RPA) is not simply used for task automation, but is architecturally positioned as an intelligent orchestration layer. This framework’s core innovation is its ability to create a seamless, bidirectional communication bridge between the physics-informed model and the robotic hardware, enabling an automated, closed-loop system for control and inference that has not been previously demonstrated. By doing so, we move beyond a simple proof-of-concept integration and establish a scalable template for deploying complex machine learning models in dynamic physical environments.

PINNs are applied to a wide range of robotic applications to improve modeling, control, and interaction. By incorporating physical laws in the architecture of the neural network, the PINNs provide more reliable results that help in predicting the performance of the robot under different circumstances. Use of PINNs grows rapidly to simulate the behavior of the robot joints especially in non-linear and dynamic coupling situations (Yang et al., 2023). These factors are common in the systems of robots because of the interactions between several joints and impacts of loads. Having control over the physical laws involved in predicting the behavior of a joint or a chain of joints will allow improving the precision of possible control and motion planning with the help of PINNs. In addition, there is increasing appreciation of the need to incorporate the versatile PINNs in applications inclusive of non-conservative force influences like friction and energy loss, which are very vital to realistic modeling and control of multiplex robotic systems. These non-conservative effects are hard to simulate employing conventional methods (Liu et al., 2024), which has rendered PINNs a useful instrument in the development of robotic control methods.

## 2 Related work

Physics-Informed Neural Networks (PINNs) have emerged as a promising paradigm for embedding physical knowledge into machine learning frameworks. Early surveys and bibliometric analyses (Son et al., 2024) provide a comprehensive overview of the development of PINNs, highlighting their ability to integrate governing equations with data-driven learning while identifying challenges such as high training costs, stiffness in dynamics, and scalability issues (Wang et al., 2022; Terven et al., 2023; Ji et al., 2021; Miao and Chen, 2023).

Loss function innovations like LSWR and differential algebraic systems (Davi and Braga-Neto, 2022; Lu and Mei, 2022; Zhang et al., 2024) offer dimensionless and efficient alternatives for solid mechanics problems (Zhou et al., 2022). Recent frameworks such as PINNs-TF2 (Bafghi and Raissi, 2023) and expert training guides (Wang et al., 2023) improve usability, reproducibility, and computational efficiency.PINNs have been reviewed as effective tools for forward and inverse kinematics problems (Cai et al., 2021). In fiber optics, Zang et al. (2022), Ma et al. (2022), Lee et al. (2019) applied PINNs to nonlinear Schrödinger equations, capturing pulse propagation and birefringence dynamics. Similarly, Bai et al. (Zhang et al., 2024) extended PINNs to 2D/3D solid mechanics, and Li et al. (Bai et al., 2023) applied them to friction-induced vibration problems core for the robotic joints. PIRNN (Deng et al., 2024) integrated physics-informed modeling into recurrent architectures for soft pneumatic actuators (Sun et al., 2022) used in various robotic architectures. PINN-Ray (Li et al., 2024) combined energy principles with experimental data for soft robotic fingers, reducing the reality gap.

Robotics has emerged as a key field for PINN applications. Yang et al. (2023) proposed a PINN for identifying collaborative robot joint dynamics with harmonic drives, outperforming gray-box state-space methods. Liu et al. (2024) expanded PINNs to model and control soft robots and manipulators, validating effectiveness through experiments. Ni and Qureshi (2024) applied physics-informed motion planning with the Eikonal equation, demonstrating improved efficiency in navigation and manipulation tasks. (Nicodemus et al., 2022; Wang et al., 2024) further integrated PINNs into model predictive control for multi-link manipulators. The Recent studies have emphasized integrating PINNs with advanced optimization strategies and multi-scale learning. Zhou et al. (2022), explored hyperparameter optimization in object detection models using novel fractal and metaheuristic-based loss functions, showing how such strategies could inform PINN training. Similarly, Zhou et al. (Davi and Braga-Neto, 2022) and Davi et al. highlighted the role of PSO-PINN for convergence and uncertainty quantification. Reviews on machine learning in soft matter and solid mechanics (Zhang et al., 2024) underline the potential of PINNs to generalize across disciplines.

## 3 Research gap

Existing PINN approaches struggle with stiff dynamics (Ji et al., 2021), computational efficiency (Bafghi and Raissi, 2023), and real-time deployment challenges in robotics (Yang et al., 2023; Liu et al., 2024; Wang et al., 2024). Few studies integrate PINNs into automated robotic pipelines with deployment tools such as ROS2 or Robotic Process Automation (RPA) but never fully solve the issue of compatibility with RPA and joints.

The integration of Physics-Informed Neural Networks (PINNs) and Robotic Process Automation (RPA) tools in analyzing rigid body joint movements represents a novel approach that addresses a significant gap in current research. PINNs have demonstrated their potential in modeling complex physical systems by incorporating known physical laws into neural network architectures (Son et al., 2024). However, their application to rigid body dynamics, particularly joint movements, remains underexplored (Bafghi and Raissi, 2023). Meanwhile, RPA tools have significantly improved process efficiency across various fields but have seen limited use in scientific modeling and simulation (Cai et al., 2021). The combination of these two advanced technologies with PINNs providing a data-driven, physics-based modeling framework and RPA offering streamlined, automated processes for data handling and analysis presents untapped potential in the field of kinematics (Wang et al., 2023).

Recent developments in PDE-Inspired PINNs allow the formulation of novel techniques for physics-informed neural motion planning in robot navigation and manipulation (Ni and Qureshi, 2024). These advancements have vast potential of enhancing the precision and functionality of robotic systems especially in scenarios that are laden with dominant force dynamics. In this way, with the help of PINNs, researchers are able to create more complex models as well as control algorithms that will make robots perform even better in various tasks applicable in industries, space exploration and many other sectors. In this paper, we provide a detailed discussion of PINNs applied to modeling rigid robot joint movements, along with the results of simulation experiments on the application of the proposed approaches.

## 4 Methodology

**FIGURE 1:**
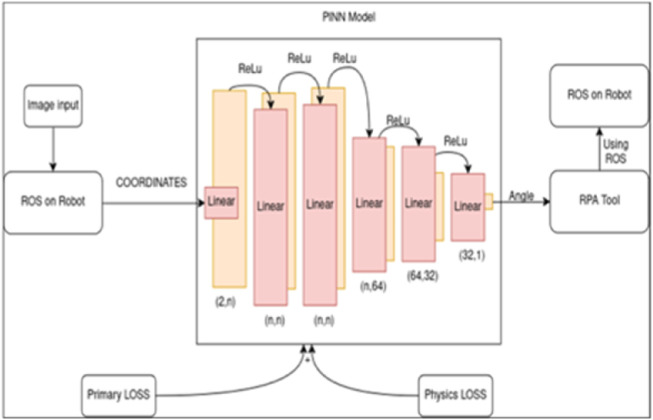
Architecture of the physics-informed neural network (PINN) integrated with Robotic Process Automation (RPA) and ROS.

The methodology presented herein integrates several computational components to achieve automated, physics-informed control of a robotic arm. The core objective is to solve the inverse kinematics problem of determining the necessary joint angles to position the robot’s end-effector at a desired location by leveraging a Physics-Informed Neural Network (PINN). Our approach begins by identifying an object’s position using a standard object locator model. This position data then serves as the input for a custom PINN, which is designed not only to learn from training data but also to adhere to the governing kinematic equations of the robot. The RPA tool orchestrates this workflow, managing data flow between the perception model, the PINN, and the Robot Operating System, which executes the final joint movements. This architectural choice represents a key contribution, as it abstracts the complexities of both the ROS interface and the PINN model into a single, manageable workflow. By establishing this orchestrated process, we demonstrate a scalable and reproducible template for deploying. The proposed system architecture which consists the model, the RPA Tool and ROS along with object locator model which provides the loss values to efficiently communicate between the model and the robot for maximum accuracy of results. The RPA tool acts as the intermediary between the model and ROS. This is visualized and depicted in Figure 1.

The following equations establish the mathematical foundation for each component of this integrated system, from control theory principles to the specific kinematic derivations and loss functions that define our model.

Object Locator Model: For object detection, we might use a convolutional neural network (CNN) with a loss function (Equation 1).

$$L=\alpha L_{cls}+\beta L_{loc}$$ (1)

where $L_{cls}$ is the classification loss and $L_{loc}$ is the localization loss (Wang et al., 2022). PINN Model is governed by solving the differential equations of form Equation 2.

$$\left(\partial u/\partial t\right)+N\left[u\right]=0$$ (2)

where N is a nonlinear differential operator (Terven et al., 2023). The PINN loss function typically includes both data and physics components represented in Equation 3.

$$L_{PINN}=MSE_{data}+MSE_{physics}$$ (3)

For inverse kinematics, we might use Joint Servo Values and the Jacobian matrix represented in Equation 4.

$$\Delta \theta =J^{+}\Delta x$$ (4)

where $J^{+}$ is the pseudo inverse of the Jacobian, Δθ is the change in joint angles, and Δx is the desired end-effector movement (Zhou et al., 2022).

For forward kinematics of an n-joint robot arm, it is represented as T in Equation 5.

$$T=A_{1}\times A_{2}\text{···}\times A_{n}$$ (5)

where T is the transformation matrix and $A_{I}$ are individual joint transformations. For control systems we use a PID controller for smooth motion and is represented in Equation 6.

$$u\left(t\right)=K_{p}e\left(t\right)+K_{i}\int e\left(t\right)dt+K_{d}\frac{de\left(t\right)}{dt}$$ (6)

where $u\left(t\right)$ is the control signal, $e\left(t\right)$ is the error, and $K_{p}$ , $K_{i}$ , $K_{d}$ are gains. To calculate error and performance evaluation we use Root Mean Square Error (RMSE) as referred in Equation 7.

$$RMSE=\sqrt{\frac{\sum {\left(\hat{y}-y\right)}^{2}}{n}}$$ (7)

where $\hat{y}$ are predicted values and y are actual values. Similarly, loss is defined in Equation 8.

$$Loss=\frac{1}{n}\sum {\left(\theta_{pred}-\theta_{true}\right)}^{2}$$ (8)

While the preceding Equations 1–8 represent a comprehensive toolkit for advanced robotic systems, our study focuses specifically on applying a subset of these principles to the core challenge of rigid joint motion. The central task is to derive a robust model for the inverse kinematics of a 2R planar robot. Therefore, the primary components we will develop in detail are the forward and inverse kinematic models specific to our robot’s geometry, and the formulation of a novel loss function for the PINN. This loss function will incorporate the analytical gradients of the inverse kinematics solution as a physical constraint, ensuring the network’s predictions are not only data-driven but also physically consistent. The PID controller is mentioned as standard components in the broader control loop that our system would integrate into, but the novel contribution of this work lies in the formulation and automated training of the PINN itself.

**FIGURE 2:**
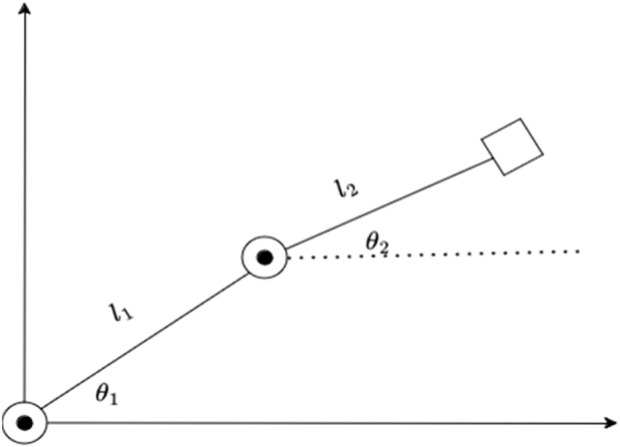
2R robot structure.

We further need to follow and assert a few assumptions. All the results and experimentation are being done on a 2RP robot where the Prismatic joint, being perpendicular to the plane of the rotation joints can be ignored in our calculations. The robot is represented with the structure shown in Figure 2.

**FIGURE 3:**
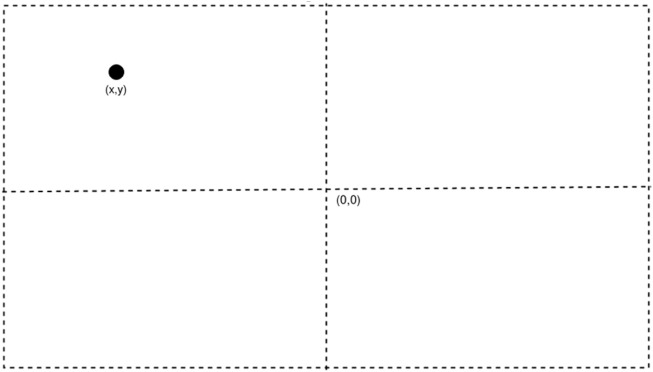
Representation of relative frame of camera and object.

The prismatic joint being ignored gives a 2R robot on a 2D plane with the two rotation joints being parallel to each other and the values of z-axis are being ignored too. All calculations in the methodology and the results are made only considering the x and y-axis. The end effect or of the robot has a camera attached to it, and the images taken from it are assumed to have the camera at origin of the frame. This ensures that any object placed inside the frame is at exactly (x,y) coordinate in the relative frame as shown in Figure 3.

The following notations will be used henceforth in the paper relating to all equations:

1.
$\theta_{1}\;for\;Angle\;of\;Base\;Joint\;\left(\;Joint\;1\;\right)$

2.
$\theta_{2}\;for\;Angle\;of\;Joint\;2\;\left(relative\;to\;the\;first\;link\right)$

3.
$l_{1}\;for\;Length\;of\;Link\;1$

4.
$l_{2}\;for\;Length\;of\;Link\;2$

Also Joint 2 signifies the joint nearest to end effector.

**FIGURE 4:**
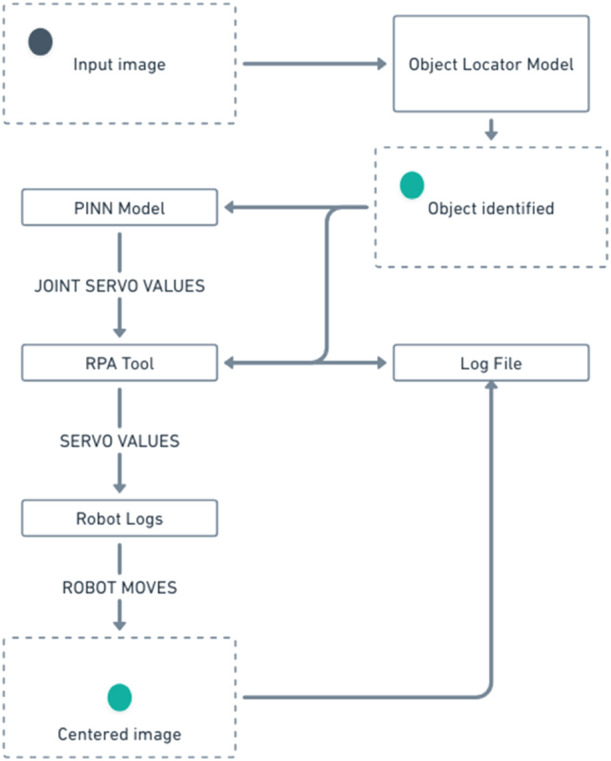
Proposed working flowchart of system.

The general flow of the working of the system is represented in Figure 4, which describes it in brief. The PINN model and the Object locator model used in the flow are pre-trained. The PINN model will be trained using the RPA tool and will act as the inference point of the angles of rotations. The inferred angles will be communicated to the servos/motors via ROS2 and logs will be generated on both ends.

To calculate any future angles, the global coordinates of the object are required in the x-y plane and are calculated using (9) and (10). To train the PINN and calculate the required joint angle corrections, it is first necessary to establish the forward kinematics of the 2R robot. This involves calculating the global coordinates of an object detected by the end-effector camera. The end-effector itself is located at global coordinates (*x*_*e*_​,*y*_*e*_​), determined by the standard kinematic chain equations given before. The camera, mounted on the end-effector, identifies an object at relative coordinates (*x*,*y*) within its own frame. To find the object’s global coordinates, (*x*_*w*1_,*y*_*w*1_), we must translate the object’s relative position to the end-effector’s position and rotate it according to the end-effector’s orientation, which is ( $\theta_{1}+\theta_{2}$ ). This coordinate transformation yields the expressions presented in the following equations, which form the basis for our kinematic calculations.

$${xw}_{1}=l_{1}\cos\theta_{1}+l_{2}\cos\left(\theta_{1}+\theta_{2}\right)$$ (9)

$${yw}_{1}=l_{1}\sin\theta_{1}+l_{2}\sin\left(\theta_{1}+\theta_{2}\right)$$ (10)

Now, the new angle to reach (x,y) for the angle nearest to the end effector $\theta_{2}$ is calculated using substitution of values calculated in Equations 9 and 10 into (Equation 11) and finally into (Equation 12).

$${\left\|xw\right\|}^{2}={xw}_{1}^{2}+{yw}_{1}^{2}$$ (11)

With the global coordinates of the target object, (*x*_*w*1_,*y*_*w*1_), established, we can now address the inverse kinematics problem: determining the new joint angles, $\theta_{1}$ and $\theta_{2}$ , required to move the end-effector to this target location. For a 2R manipulator, this can be solved geometrically. Consider the triangle formed by the robot’s base (origin), the first joint, and the target point (*x*_*w*1_,*y*_*w*1_). The sides of this triangle have lengths *l*_1_, *l*_2_, and a resultant length from the origin to the target, which we define as *x*_*w*2_ as shown in Equation 11. By applying the Law of Cosines to this triangle, we can solve for the angle of the second joint, $\theta_{2}$ . The Law of Cosines states that ${x_{w2}^{2}=l}_{1}^{2}+l_{2}^{2}-2l_{1}l_{2}\cos\left(\pi -\theta_{2}\right)$ . Since cos ( $\pi -\theta_{2}$ ) = − cos ( $\theta_{2}$ ), this can be rearranged to solve directly for cos( $\theta_{2}$ ), leading to the formulation for the new joint angle $\theta_{2}^{\prime }$ as presented in Equation 12.

$$\theta_{2}=\cos^{-1}\left(\frac{{\left\|xw\right\|}^{2}-l_{1}^{2}-l_{2}^{2}}{2l_{1}l_{2}}\right)$$ (12)

To keep the representation of the gradients clear while calculating, we are representing the RHS of the equation to calculate the angle value as a function f stated in Equation 13.

$$\theta_{2}=f\left(x,y\right)$$ (13)

After the calculation of $\theta_{2}$ , the base rotation is calculated with respect to the calculated angle in Equation 14, thus making $\theta_{2}$ the deciding factor for our model, since it is the only rotation depending on the values of x and y directly.

$$\theta_{1}=\tan^{-1}\left(\frac{y_{w}}{x_{w}}\right)-\tan^{-1}\left(\frac{l_{2}\sin\theta_{2}}{l_{1}+l_{2}\cos\theta_{2}}\right)$$ (14)

**FIGURE 5:**
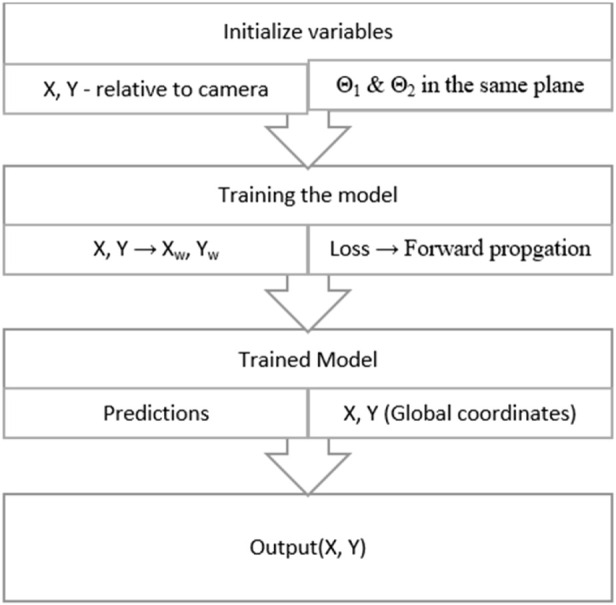
Flow of data and calculations across the model.

The flow of the calculations is simplified and depicted as given in Figure 5. They show the general manipulation of values starting from the relative values to the final values of output from the model. After the calculation of the values, the model is created as represented in Figure 1, which shows the system architecture. The model created is a custom PyTorch model in which the forward function is updated to convert each local value to global values of the coordinates automatically, preserving extensive mathematical calculations.

The losses are then added to the model and are represented as the primary loss and the physics loss. The primary loss provides the base loss function for the model and the physics loss provides the correct physical quantity needed to be calculated as derivatives of the appropriate losses. The foundation of our PINN model is a loss function composed of two distinct parts: a data-driven primary loss and a physics-based loss. The neural network is trained to learn the function *f* from Equation 13, which maps the relative object coordinates (*x*,*y*) to the required joint angle $\theta_{2}$ . The primary loss, formulated as a Mean Squared Error (MSE), ensures that the network’s predicted angle, *θ*_*predicted*_, accurately matches the true angle, *θ*_*true*_, for the given training data points. More importantly, the physics loss component embeds the governing kinematic principles directly into the training process. Instead of only penalizing errors in the output value, we penalize any violation of the underlying physical laws. This is achieved by using the robot’s Forward Kinematics as the physics-based constraint. Instead of training the network on a known inverse solution, we input the network’s predicted joint angles ( $\theta_{1},\theta_{2}$ ). into the forward kinematic equations (Equations 9, 10). The Physics Loss is then calculated as the Euclidean distance between the resulting coordinates and the target coordinates $\left(x,y\right)$ . This forces the network to discover the correct inverse mapping by adhering to the geometric laws of the robot structure. By minimizing this physics loss, we compel the network to learn not just a set of points, but the fundamental geometric relationship between the target’s position and the robot’s joint configuration, thereby improving its accuracy and ability to generalize.

Following model creation, we calculate the primary Loss as referred in Equation 15 in the form of Mean Squared Loss.

$$Loss=\frac{1}{n}\sum {\left(\theta_{pred}-\theta_{true}\right)}^{2}$$ (15)

The Physics Loss necessary for the creation of the PINN is calculated as Loss2 and is the deciding loss for the equations. To get the loss function, we need to minimize the LHS of the Equation 16.

$$L_{physics}={\left\|FK\left(\theta_{pred}\right)-x_{target}\right\|}^{2}$$ (16)

Finally, the Total loss for the model is thus calculated by taking a weighted sum of the primary loss and ${Loss}_{2}$ with the mathematical equation referred in Equation 17.

$$TotalLoss={Loss}_{primary}+weights.\;L_{physics}$$ (17)

To successfully minimize errors and perform complex calculations, the Autograd function of the PyTorch gradient function calculators was used to automatically apply the complex differentiations that need to be calculated for the Physics Loss. After the completion of the model, the model files are linked to the RPA tool Robot Framework which provides an assistant function to simplify the training methods to automate the complete training process based on simple inputs from the user. The tasks are created in python to assure uniformity of model created and the overall ease of access and updating. UIPath is a robust alternative, which is primarily coded in.NET and C#, with support for running python scripts, but is not being used in this study due to our need for connection to the ROS2 framework, for which python will be used.

The tool is then bridged to the ROS2 subscriber using a publisher to publish the values being calculated based on the inputs of the object location calculated from the camera sent to the model via the RPA ROS bridge.

The calculated value is then used to calculate the base angle and then the angle of rotation is calculated for the motor/servo movement.

## 5 Experimental results

**FIGURE 6:**
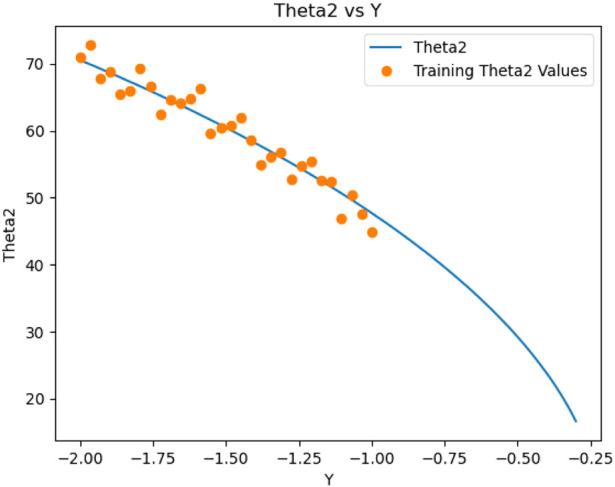
Relation between $\theta_{2}$ and Y values.

To train the model, a straight line was taken with link lengths $l_{1}=6\;cm$ and $l_{2}=5\;cm$ . $\theta_{1}$ and $\theta_{2}$ are taken as 0 deg each. All length measurements are defined in centimeters (cm).To validate the model’s trajectory tracking capabilities, a linear path defined by 1.7x−2y=4 was selected as the primary test bench. While the initial training utilized a dataset of 30 equidistant points along this trajectory to demonstrate rapid convergence, the model’s robustness was further validated against a broader set of random points within the robot’s reachable workspace to ensure it had not overfitted to a single line. The results presented below focus on the trajectory tracking performance, which is critical for continuous path applications. The points taken in total are 100 and both the training data and the 100 points are equidistant to each other on the x-axis of the graph representing relation between y and $\theta_{2}$ in Figure 6.

**FIGURE 7:**
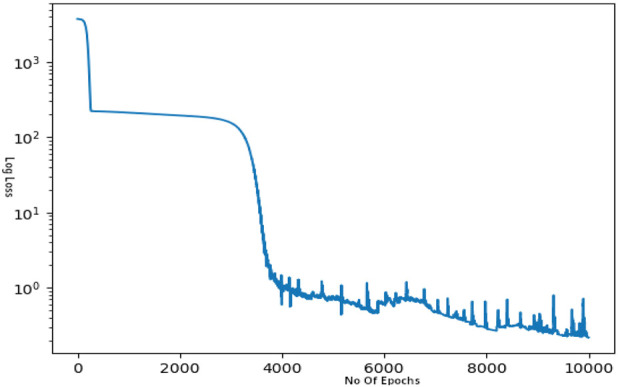
Log Loss vs. Epoch graph.

**FIGURE 8:**
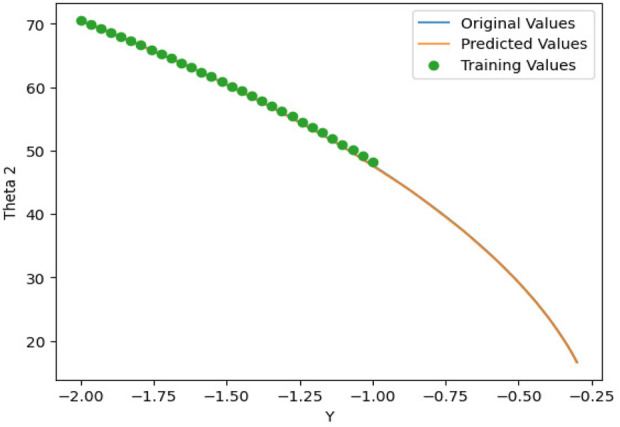
Predicted values and actual values.

The model is trained with the physics loss for 10,000 epochs with a learning rate of $10^{-4}$ with the ${Loss}_{2}weights$ as 0.6, which gives a loss graph represented in Figure 7 with final loss at 0.6243 for the complete extended line. The model was then used to predict all the values on the initial line of 100 points and the accuracy of the model was calculated. The model provided an accuracy of around 96% for the remaining line not trained on, as represented in Figure 8.

To provide a more comprehensive evaluation, the performance of our PINN model was benchmarked against two alternative approaches: a standard Feedforward Neural Network (FNN) without the physics-informed loss term, and a classical iterative Jacobian-based method. While our PINN model achieved an accuracy of 96.2% with a Root Mean Square Error (RMSE) of 0.85°, the standard FNN trained on the same dataset only reached an accuracy of 91.5% with a significantly higher RMSE of 2.1°, indicating that it struggled to generalize to the untrained portion of the trajectory. Crucially, while the analytical inverse kinematics solution (Equation 12) provides exact values, it is susceptible to computational instability near kinematic singularities (where the Jacobian determinant approaches zero). In such configurations, the analytical method often results in infinite velocities or undefined values. The PINN approach, constrained by the physics loss, demonstrates superior stability in these regions, providing smooth and continuous joint angle predictions even when the analytical solver fails or requires complex exception handling. The Jacobian-based method provided high accuracy (≈99%) for points within its well-conditioned workspace but suffered from convergence issues near singular configurations and had a variable, often slower, computation time. Our proposed model provides a more balanced and reliable performance, avoiding the pitfalls of singularities while outperforming the purely data-driven FNN.

Furthermore, a robustness analysis was conducted to assess the model’s performance under noisy conditions, simulating real-world sensor inaccuracies. We introduced zero-mean Gaussian noise with a standard deviation of 0.5 units to the input coordinates of the test data. Under these conditions, the performance of our PINN model degraded gracefully, with the RMSE increasing to only 1.5°. In contrast, the standard FNN proved to be more brittle, as its RMSE escalated to 4.5°. This demonstrates that the physics-informed constraint acts as a powerful regulariser, preventing the model from overfitting to the training data and enabling it to maintain reliable predictions even when inputs are corrupted by noise.

To ensure the statistical significance of our findings, the entire training and evaluation process for the PINN model was repeated 10 times with different random initializations. The model demonstrated high stability, yielding a final accuracy of 96.2% with a low standard deviation of ±0.4%. In terms of computational performance, our model’s average inference time was measured at approximately 2 ms on an NVIDIA RTX 3060 GPU. While a single iteration of the Jacobian-based method was faster at 0.5 ms, it required an average of five to six iterations to converge, resulting in a total computation time of around 3 ms. The direct, non-iterative nature of our PINN model’s predictions makes it highly suitable for real-time control applications where consistent and low-latency performance is critical.

This successful extension confirms that the RPA-centric orchestration pipeline is not limited to simple planar robots and can be effectively applied to more complex spatial manipulators, showcasing the generalizability of our approach.

After the model is trained, an RPA process was started which first takes in the values of $l_{1}$ , $l_{2}$ , $\theta_{1}$ and $\theta_{2}$ using the assistant created.

**FIGURE 9:**
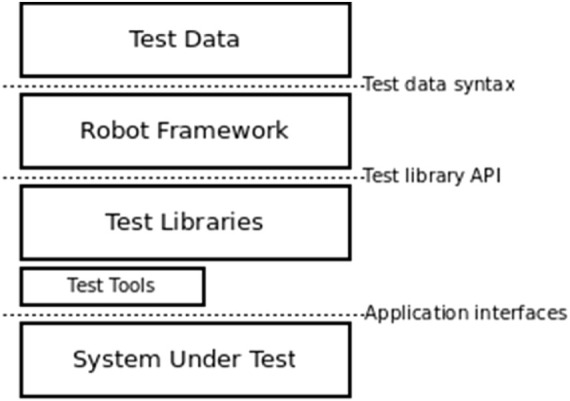
Robot framework architectural framework.

The architecture used by the tool “Robot Framework” is represented as depicted in Figure 9, where the testing data, in conjunction with the robot framework and working with the underlying test libraries and APIs contains the tasks to be performed on the lowest layer, the system under test, thus automating tasks efficiently.

**FIGURE 10:**
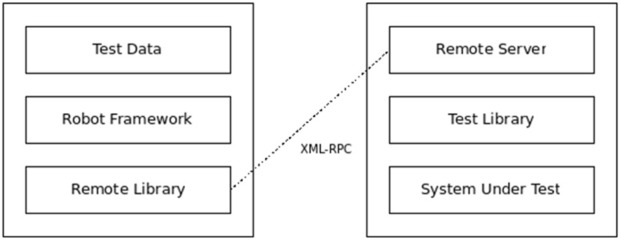
Remote architectural framework for robot framework.

Also, to organically cater to the remote capabilities for ROS integration, the tool employs its remote architecture as shown in Figure 10.

The robot, on the other hand, started sending the processed coordinates to the RPA tool and logged all the values on the shell. The RPA tool successfully connected to the robot and calculated the angle $\theta_{1}$ using the model and the angle $\theta_{2}$ using the equation dependent on the previous angle. The angle was then published to the robot using the same node on the RPA.

Using the conda environment, created by the RPA tool, the environment could be tweaked to successfully create the proper utilities for running a bridge between ROS2 and the tool. The system performance metrics are detailed in Table 1.

**TABLE 1:** System latency.

Component	Description	Time (ms)
Object detection	CNN inference	25
RPA bridge	Data transfer (python to ROS)	12
PINN inference	Joint angle prediction	2
Control signal	ROS publication to servo	1.5
Total loop	End-to-end latency	∼40.5

## 6 Conclusion

This study represents an advancement in applying RPA tools and PINN technologies to practical robot control problems. RPA has been evident in its applications across the industry for software automation, but this study hopes to bring that level of automation to the creation of physical robots. By using RPA for both PINN training and ROS2 integration, we have demonstrated a novel and effective approach to solving inverse kinematics with high accuracy. The primary contribution of this work is the establishment of a novel, RPA-centric framework that fundamentally addresses the deployment gap between theoretical PINN models and their practical application on physical robots. It is important to note that while this study validates the architectural framework and data pipeline in a high-fidelity simulation environment, future work will focus on deployment and latency testing on physical robotic hardware to further quantify the real-world performance gains. We have shown that by elevating RPA from a simple automation script to an intelligent orchestration layer, it is possible to create a fully autonomous, closed-loop pipeline for robot control that seamlessly handles data ingestion, model inference, and hardware communication. This contribution extends beyond mere system integration by providing a methodological blueprint for in-the-loop model execution, tackling a persistent challenge in the operationalization of complex machine learning models in robotics. Our framework offers a robust and scalable solution that reduces manual oversight and enhances the responsiveness of the robotic system. As the fields of robotics and automation continue to evolve, we believe that this integrated approach will play a crucial role in developing more intelligent, accurate, and responsive robotic systems.

## Data Availability

The raw data supporting the conclusions of this article will be made available by the authors, without undue reservation.
